# Supplementary figures and images for: CD14 and CD26 from serum exosomes are associated with type 2 diabetes, exosomal Cystatin C and CD14 are associated with metabolic syndrome and atherogenic index of plasma
Source: PeerJ. 2022 Jul 12;10:e13656. doi: 10.7717/peerj.13656 (PMC9285478; doi:10.7717/peerj.13656)

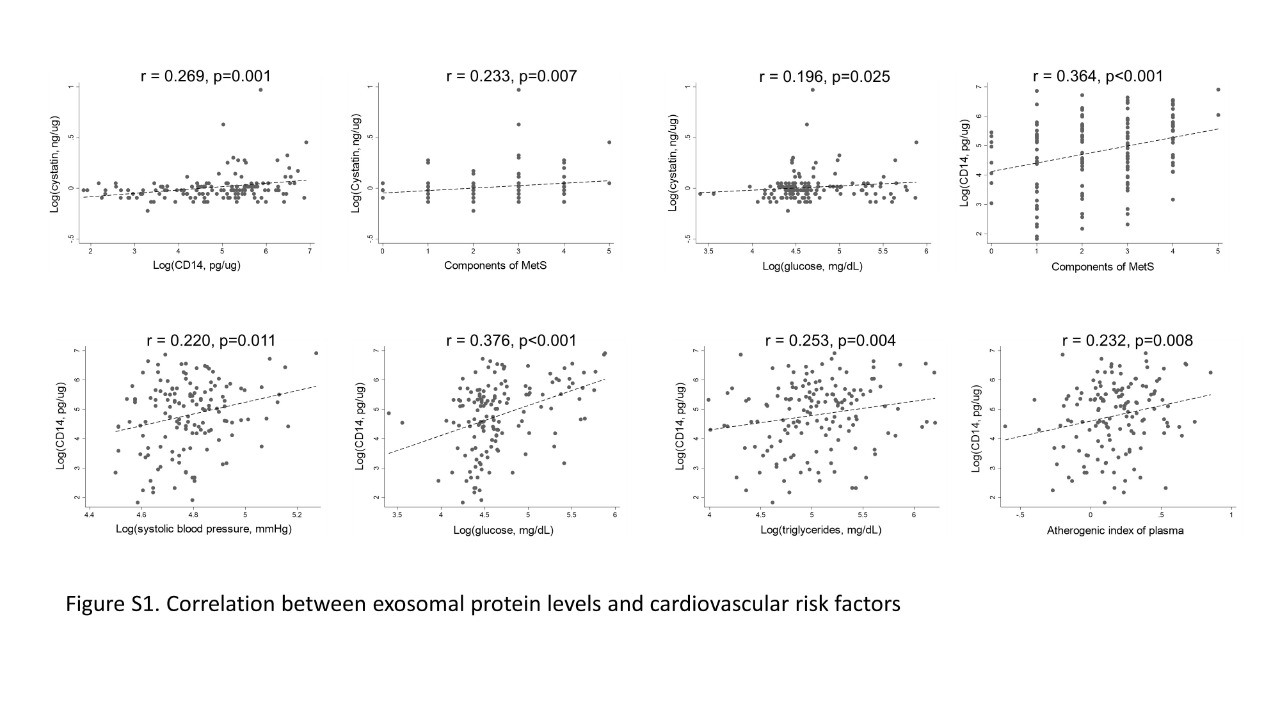

Supplement: Figure S1 [file peerj-10-13656-s001.jpeg]

## CD9

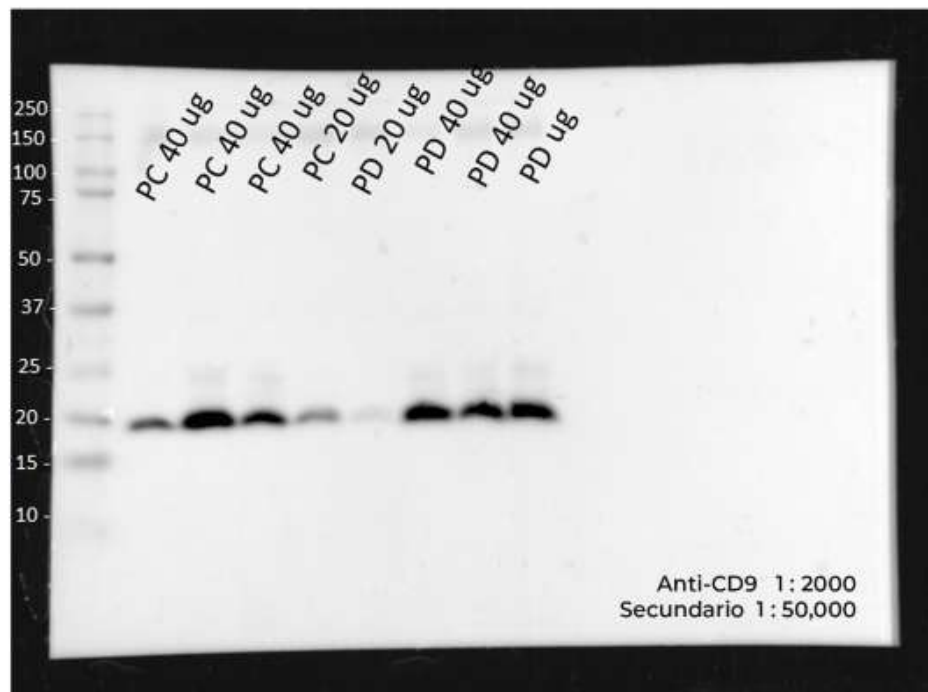

## CD81

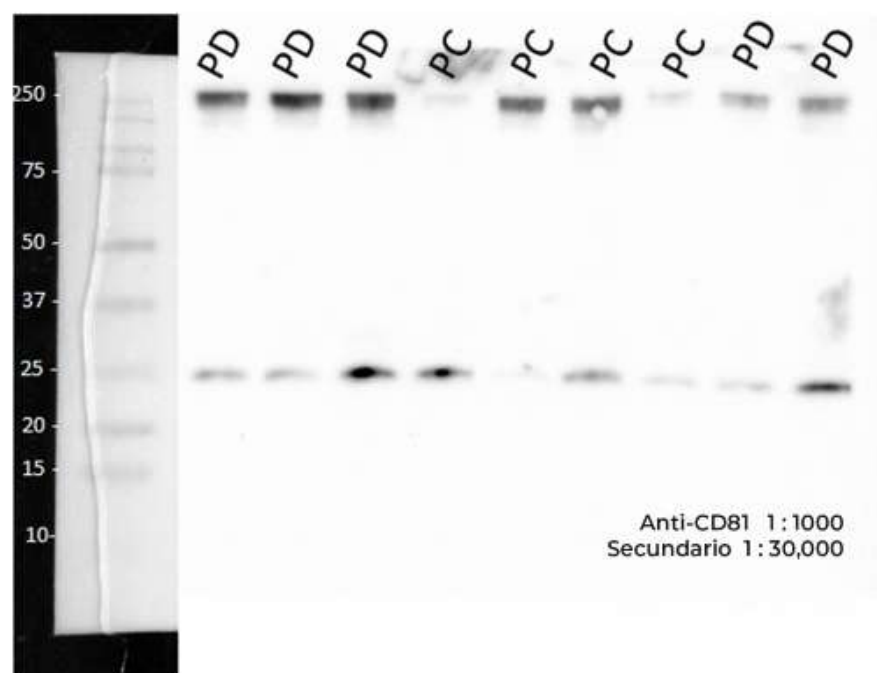

## CD63

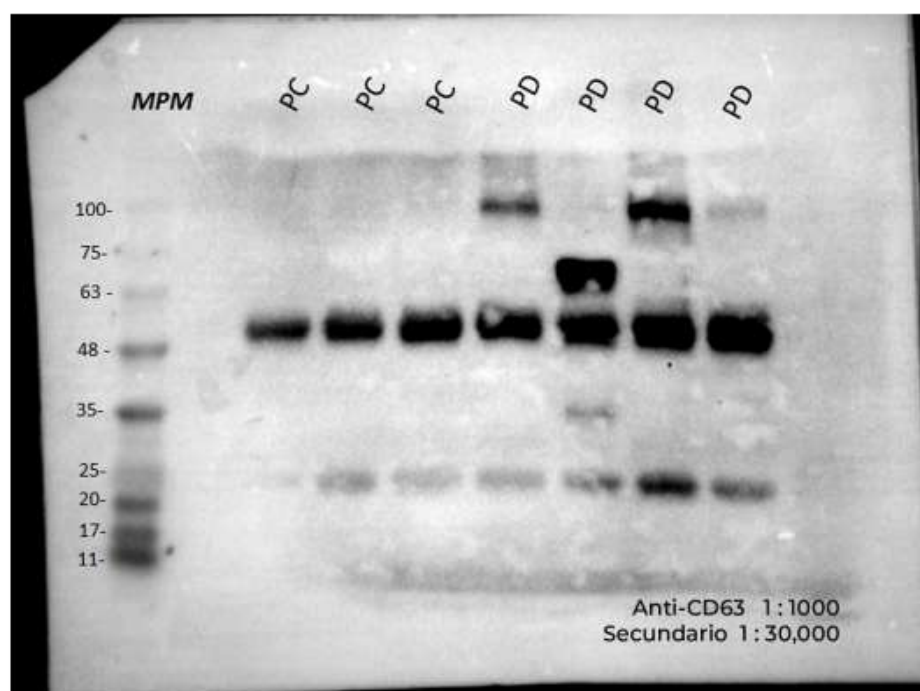

Supplement: Supplemental Information 2 — Uncut and unedited western blot membranes, directly obtained from the Biorad imaging system, the photos include the detection of CD9, CD81 and CD63 proteins in exosomes from the serum of diabetic people and control people [file peerj-10-13656-s002.pdf]

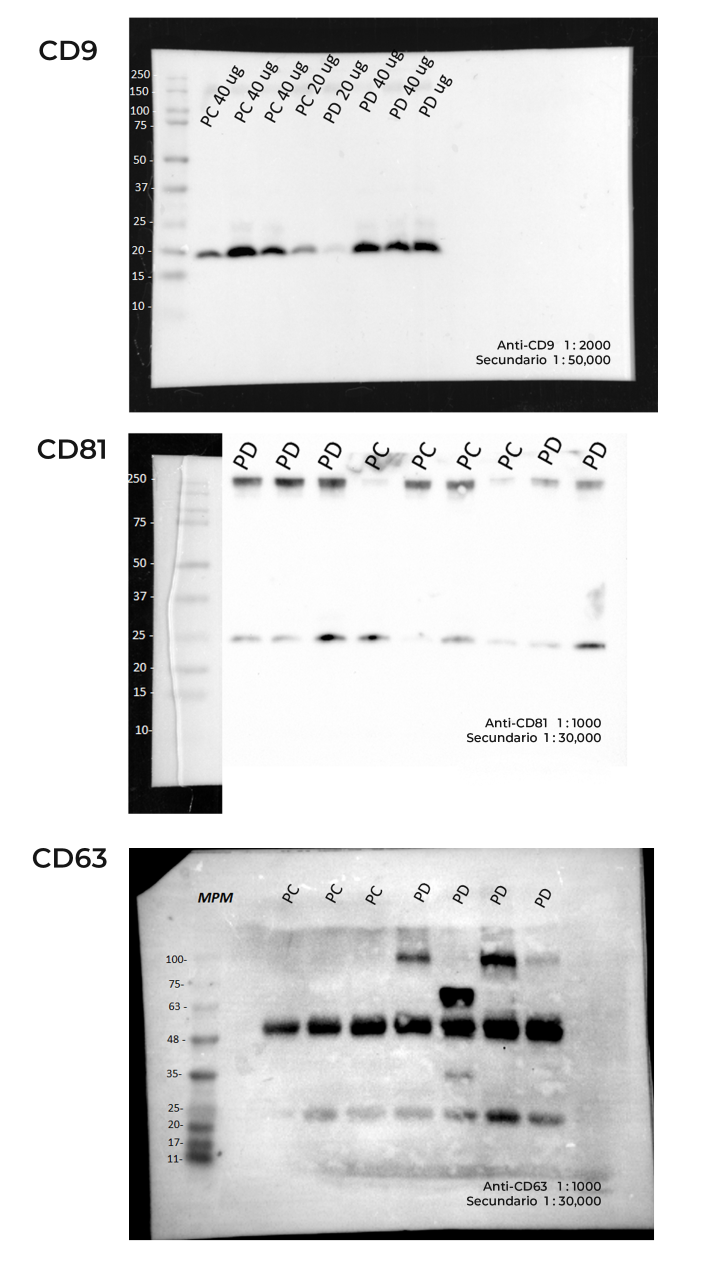

Supplement: Supplemental Information 3 — Uncut and unedited western blot membranes, directly obtained from the Biorad imaging system, the photos include the detection of CD9, CD81 and CD63 proteins in exosomes from the serum of diabetic people and control people [file peerj-10-13656-s003.png]
